# Stabilization of a Broadly Neutralizing Anti-Chikungunya Virus Single Domain Antibody

**DOI:** 10.3389/fmed.2021.626028

**Published:** 2021-01-28

**Authors:** Jinny L. Liu, Emily M. Webb, Dan Zabetakis, Crystal W. Burke, Christina L. Gardner, Pamela J. Glass, Patricia M. Legler, James Weger-Lucarelli, George P. Anderson, Ellen R. Goldman

**Affiliations:** ^1^U.S. Naval Research Laboratory, Center for BioMolecular Science and Engineering, Washington, DC, United States; ^2^Virginia Polytechnic Institute and State University, Blacksburg, VA, United States; ^3^Virology Division, U.S. Army Medical Research Institute for Infectious Diseases, Fort Detrick, MD, United States

**Keywords:** chikungunya virus, old world, new world, alphavirus, neutralization, melting temperature, single domain antibody

## Abstract

A single domain antibody (clone CC3) previously found to neutralize a vaccine strain of the chikungunya virus (PRNT_50_ = 2. 5 ng/mL) was found to be broadly neutralizing. Clone CC3 is not only able to neutralize a wild-type (WT) strain of chikungunya virus (CHIKV), but also neutralizes WT strains of Mayaro virus (MAYV) and Ross River virus (RRV); both arthralgic, Old World alphaviruses. Interestingly, CC3 also demonstrated a degree of neutralizing activity against the New World alphavirus, Venezuelan equine encephalitis virus (VEEV); albeit both the vaccine strain, TC-83, and the parental, WT Trinidad donkey strain had PRNT_50_ values ~1,000-fold higher than that of CHIKV. However, no neutralization activity was observed with Western equine encephalitis virus (WEEV). Ten CC3 variants designed to possess a range of isoelectric points, both higher and lower, were constructed. This approach successfully identified several lower pI mutants which possessed improved thermal stabilities by as much as 10°C over the original CC3 (T_m_ = 62°C), and excellent refolding abilities while maintaining their capacity to bind and neutralize CHIKV.

## Introduction

Chikungunya fever is a reemerging infectious disease caused by the chikungunya virus (CHIKV), a mosquito-borne alphavirus. Old World alphaviruses typically result in persistent, or recurring, arthralgia after acute infection (1, 2), while the more virulent New World alphaviruses can cause lethal encephalitis (3). In late 2013, CHIKV emerged in the Americas where it has caused millions of human infections (4). Neutralizing antibodies have shown promise as both prophylactic and therapeutic agents against CHIKV (5). To date, both polyvalent immunoglobulin (Ig) and monoclonal antibodies (mAbs) have been studied (6, 7). Some mAbs have been reported to be broadly neutralizing, being effective against CHIKV and several other arthralgic Old World alphaviruses (8).

Recombinantly expressed antibody binding domains, such as single domain antibodies (sdAb), offer an alternative format for antiviral therapeutics (9). Comprised of the variable domain of unconventional heavy-chain only antibodies found in camelids, sdAb function as small and robust recognition elements with affinities comparable to those of conventional IgG (10, 11). Advantages of sdAb over conventional immunotherapeutics include their ability to access cryptic epitopes, low molecular weight, and ease of production in *E. coli* (12, 13). Several sdAb have been tailored to a variety of specific applications such as protease resistance (14), ability to function in the presence of denaturants (15, 16), and have the ability to maintain their antibody-antigen complexes even at elevated temperatures (17, 18). Fast clearance, although a potential drawback, can be overcome through strategies such as PEGylation or genetic fusion with an anti-albumin sdAb or Fc-domains (19–21).

Previously, we described five sdAb able to bind CHIKV virus-like particles (VLPs), or recombinant CHIKV envelope protein. Two of the clones (CC3 and CA6) were evaluated for their ability to neutralize CHIKV; whereas both clones showed neutralization, CC3 was ~80 times more effective (22). In this study, we demonstrate that CC3 can also neutralize other Old World as well as New World alphaviruses. In addition, we constructed and characterized a series of CC3 isoelectric point (pI) variants and identified mutants with improved stability and increased ability to refold after heat denaturation that retain their neutralization capability.

## Materials and Methods

Expanded methods are provided in the Supplementary Information.

### Materials

The CHIKV-specific sdAb CC3 was previously described (22). All enzymes used for cloning were from New England Biolabs (Ipswich, MA). CHIKV VLPs and recombinant envelope proteins were from the Native Antigen Company (Oxford, UK). The BSL2 CHIKV strain 181/25 was kindly provided through the World Reference Center for Emerging Viruses and Arboviruses (WRCEVA, Galveston, TX). The RRV Rarotonga strain was obtained from the U.S. Centers of Disease Control and Prevention (CDC, Atlanta, GA). The WT MAYV strain TRVL 4675 (23), WT CHIKV strain SL-15649 (24) (a gift from Dr. Mark Heise), VEEV TC-83 vaccine strain (25) (a gift from Dr. Scott Weaver), and WEEV Imperial 181 strain (26) (a gift from Dr. Aaron Brault) were rescued from infectious clones. Viral rescue was performed as described previously (23). Lassa VLPs were from Zalgene (Germantown, MD). Unless otherwise specified, common reagents were from Sigma-Millipore, VWR, or Thermo Fisher.

### Construction and Production of sdAb Variants

Genes for sdAb variants were synthesized by Eurofins Genomics (Louisville, KY) with flanking NcoI and NotI sites. Mutants were designed based on the consensus sequence of the CA6 neutralizing sdAb as well as toxin binding sdAb (22, 27). The hop tail is based on the patent application by Neal Anthony Eric Hopkins (28), and was flanked by NotI and XhoI sites. All sdAb were expressed in *E. coli* and purified as previously described (27). The amino acid sequences of the produced sdAb with the hop tail is provided in the Supplementary Information. Variants with the hop tail are denoted with the clone name followed by “hop.” Theoretical pI was determined using the on line tool ExPASy (29).

### Measuring Melting Temperatures and Binding Abilities

Thermal denaturation was monitored using circular dichroism (CD) and binding ability was assessed by MagPlex direct binding assays as previously described (22).

### Neutralization

Neutralization studies were similar to those previously described (22). Minor differences in plaque reduction and neutralization testing (PRNT) protocols between the three laboratories are detailed in the Supplementary Information.

## Results and Discussion

### Neutralization of Alphaviruses

We had previously identified five CHIKV binding sdAb. The neutralization capacity of two of the five (clones CC3 and CA6) was determined by IBT Bioservices (Rockville, MD). Both neutralized CHIKV 181/25 (22); however, CC3 was much more effective than CA6. Further testing of all five clones at the Naval Research Laboratory (NRL) and Virginia Tech (VT) confirmed that CC3 demonstrated far superior neutralization than any of the other clones identified (Supplementary Table 1). The ability of CC3 to neutralize CHIKV 181/25 also was independently verified by the US Army Medical Research Institute for Infectious Diseases (USAMRIID).

**Figure 1:**
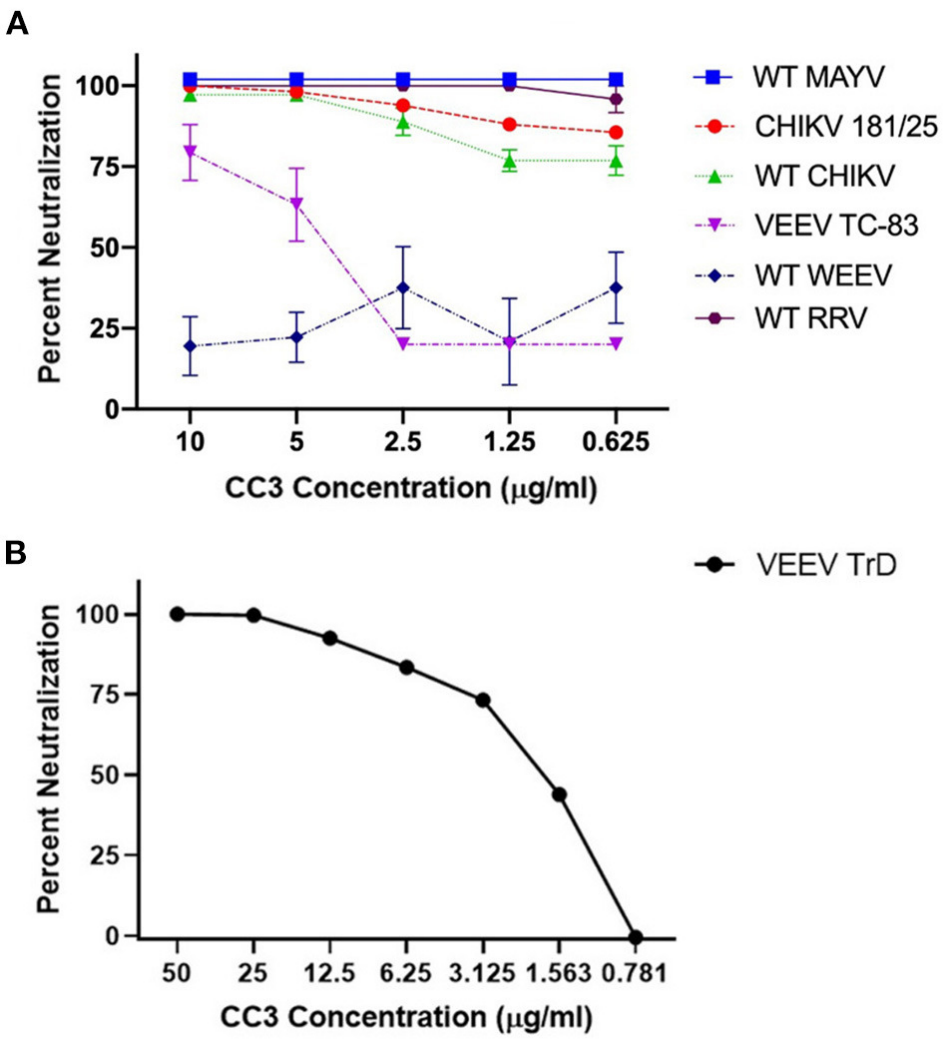
Ability of clone CC3 to neutralize the indicated alphaviruses. **(A)** Shows neutralization of WT MAYV, CHIKV 181/25, WT CHIKV, VEEV TC-83, WT WEEV, and WT RRV. **(B)** Shows neutralization of WT Trinidad donkey (TrD) strain of VEEV.

With a focus on CC3, we determined its ability to neutralize WT strains of CHIKV, MAYV, and RRV; all arthralgic, Old World alphaviruses. CC3 showed potent neutralization of each Old World alphavirus tested (PRNT_50_ values all <0.625 μg/ml; Figure 1). In addition, we examined the ability of CC3 to neutralize two New World alphaviruses: VEEV and WEEV. Interestingly, while CC3 showed some ability to neutralize both the TC-83 vaccine strain of VEEV (PRNT_50_: 4.0 μg/ml) as well as the parental WT Trinidad donkey strain (PRNT_50_: 1.9 μg/ml), no neutralization was observed with WEEV (Figure 1). A toxin binding sdAb [ACVE (30)] was run as an isotype sdAb control, and as expected no neutralization of any of the viruses was observed (Supplementary Figure 1).

### Variants of CC3

**Figure 2:**
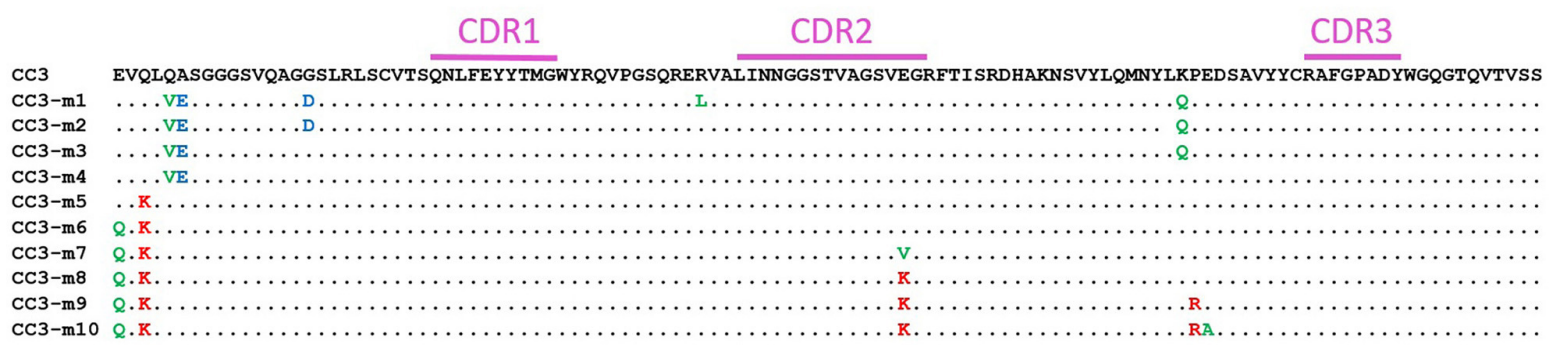
Amino acid sequence (single letter abbreviation) of CC3 and the variants. The CDR regions are indicated by a purple bar. Unchanged amino acids are indicated with a dot. Substitutions to negative amino acids are in blue, substitutions to uncharged amino acids are in green, and substitutions to positively charged amino acids are in red.

Adding charges is a known path toward stabilizing antibody binding fragments that can result in increased melting temperatures or decreased aggregation, or both. We have improved melting temperatures and refolding ability of sdAb by adding negative charge (27, 31). Others have also found that negative charge within sdAb domains is correlated with thermal stability and refoldability (32). However, adding positive charges to a conventional antibody binding domain was also shown to improve stability (33), and in a study of artificial human sdAb, the charge of the scaffold determined if the addition of negative or positively charged amino acids prevented aggregation (34). Although typically negative charges are associated with the stability of sdAb, we aimed to explore a range of charge variants. For this study, a series of ten CC3 variants were constructed. These variants have a range of pIs both higher and lower than the WT-CC3 through specific charge changes to framework residues known to accommodate such a change (Figure 2, Table 1, Supplementary Table 1).

**Table 1:** Theoretical pI, protein yield, melting temperature (T_m_), and percent refolding of the CC3-hop variants.

**Clone**	**Theoretical pI**	**Yield mg/L**	**T_**m**_ (^**°**^C)**	**% refold**
CC3-hop	7.75	12.4	62	80
CC3-m1hop	6.12	28	**76**	**95**
CC3-m2hop	6.35	18	**69**	**91**
CC3-m3hop	6.63	9.9	**72**	**91**
CC3-m4hop	7.04	9.2	**70**	**81**
CC3-m5hop	8.40	20.5	58	46
CC3-m6hop	8.75	9.6	55	38
CC3-m7hop	9.0	11	55	19
CC3-m8hop	9.18	11	56	19
CC3-m9hop	9.35	5.8	54	5
CC3-m10hop	9.51	3.4	49	15

*The T_m_ and percent refolding for the mutants with a more negative pI than CC3-hop are shown in bold. A representative protein yield is show; although variation is seen between preparations, typically very similar yields are observed. The error on Tm is ± 1°C*.

### sdAb Production

All sdAb variants were purified by immobilized metal affinity chromatography followed by gel filtration; only monomeric sdAb was used for further characterization. Each of the sdAb variants was first produced with only a hexa-histidine tag for purification (Supplementary Table 2). However, it can be advantageous to express the sdAb with residues that can be used for covalently conjugating biotin, fluorophores, or polyethylene glycol (PEG). To this end, a second version of each clone was produced with a C-terminal cysteine before the histidine tag which could be used to specifically label the sdAb with molecules containing a maleimide group. Unfortunately, the addition of the terminal cysteine to these constructs resulted in poor yields and a large dimer peak in gel-filtration (Supplementary Figure 2). The monomeric protein yields were only ~0.4–1.5 mg/L. More robust production is needed for reagents that may potentially be examined for their therapeutic potential. In an effort to enhance production yields, the “hop tail,” a peptide tag that contains a short linker and two cysteine residues plus an amino acid sequence for substrate recognition by *E. coli* disulfide isomerase was added (26). Fusions of CC3 and the hop tail significantly enhanced yields, giving between 3.4 and 28 mg/L of monomeric protein (Table 1, Supplementary Figure 2). The two most positively charged mutants (CC3-m9hop and CC3-m10hop) produced the least, with yields of 3.4 and 5.8 mg/L and had pI values of 9.51 and 9.35, respectively. Others have also observed lower yields for more positively charged variants of a sdAb (34). However, overall there was no clear correlation between pI and sdAb yield.

### Binding Ability

The CC3 variants with the hop tail were assessed for their ability to bind to CHIKV VLPs to ensure maintenance of function (Supplementary Figures 3, 4). Every mutant was able to bind the immobilized VLP; however, the most negatively-charged variant (CC3-m1hop) appeared to bind poorly. This variant contains an arginine to leucine substitution in framework 2 at a position which is frequently a leucine in sdAb, however this position has been observed to make contacts with antigen which could explain the observed reduction in binding ability (35). Variants 8, 9, and 10, contain a glutamic acid to lysine substitution at the end of complementarity determining region 2 (CDR2). In a study of sdAb sequences and structures, this position is often a lysine and was observed to be involved in antigen binding in over 10% of the structures examined (35), however no reduction in binding was observed with the CC3 variants.

Positively charged antibody binding domains have previously been correlated with higher non-specific binding (36), therefore, binding to Lassa VLPs was examined to assess specificity of the CC3 variants (Supplementary Figure 3). The most positive variant, CC3-m10, showed a modest signal of ~170 on the Lassa VLPs at the highest concentration vs. a signal of ~10,000 on CHIKV VLPs. The other variants showed no binding to irrelevant target.

### Melting Temperature and Refolding

Melting temperatures and refolding ability were assessed by CD and are shown in Table 1, Supplementary Table 2 for variants with and without the hop tail, respectively. Representative melting and refolding curves are shown in Supplementary Figure 5. The melting temperatures of the variants were not substantially affected by the addition of the hop tail, although some clones showed somewhat poorer refolding with the tail. The two most positively charged mutants were much less stable, with lower melting temperatures, and greatly reduced refolding ability compared to the original CC3. All negatively charged mutants had increased melting temperatures and superior refolding ability compared to the original clone. This is consistent with our previous observation that introducing negative charge effectively stabilizes the sdAb, increasing their melting temperatures and enhancing their ability to refold after heat denaturation (31). We previously observed that changing the 5 and 6 positions in framework 1 of a sdAb to V and E, respectively, led to an increase in melting temperature of up to 7°C (37). Separately, other researchers showed that the 5V mutation can be stabilizing (38). Because of our previous observations, we included the 5V substitution in all of our negative pI variants even though it did not contribute to the decrease in pI. Variant CC3-m4, which has only the Q5V/A6E substitutions, had a 10°C increase in its melting temperature compared to CC3 (Supplementary Table 2). Mutants CC3-m1hop, CC3-m2hop, CC3-m3hop, and CC3-m4hop all had lower pI values than the original CC3-hop and possessed a melting temperature at least 7°C higher. Each regained at least 80% of their secondary structure after heat denaturation.

### Neutralization

Clones CC3, CC3-hop, and all of the variants containing the hop tail were tested for their ability to neutralize CHIKV 181/25 (Table 2, Supplementary Figure 6). The hop tail had only a minor adverse impact on the sdAb's neutralization ability. However, the variant with the lowest pI, and the variants with the four highest pI values had reduced neutralizing activity. Due to the decreased binding ability of CC3-m1hop, the decreased neutralization was not surprising, however we had not expected decreased neutralization for the variants with high pI. All four of the positively charged clones had a change in their CDR2, which did not significantly affect their binding to VLPs, but perhaps does affect their neutralizing activity.

**Table 2:** Neutralization of CHIKV strain 181/25 by CC3, CC3-hop, and variants.

**CHIKV sdAb**	**PRNT_**50**_ (ng/mL)**	**PRNT_**90**_ (ng/mL)**
CC3	2.5 ± 0.07	31 ± 27
CC3-hop	5.4 ± 0.92	42 ± 22
CC3-m1-hop	12.3 ± 1.0	296 ± 21
CC3-m2-hop	4.6 ± 2.7	18 ± 2
CC3-m3-hop	3.9 ± 0.2	23 ± 2
CC3-m4-hop	3.8 ± 0.8	19 ± 16
CC3-m5-hop	4.4 ± 0.4	16 ± 2
CC3-m6-hop	8.9 ± 0.2	54 ± 14
CC3-m7-Hop	14.9 ± 3.5	146 ± 23
CC3-m8-Hop	21.1 ±2.3	209 ± 146
CC3-m9-hop	34.7 ± 6.8	149 ± 27
CC3-m10-hop	24.2 ± 2.0	169 ± 92

*Each measurement was done in duplicate. In addition 2–3 biological replicates were performed; the average and standard deviation between sets of experiments are reported*.

## Conclusion

We found the anti-CHIKV sdAb CC3 to be broadly neutralizing, and constructed a series of CC3-based variants to assess the correlation of pI and stability of this sdAb. Based on the combined results of binding, stability and neutralization assays, the CC3-m2hop, CC3-m3hop, and CC3-m4hop were found to be the best candidates for further study. These CC3-based constructs offer a specific site for modification (such as PEGylation) to provide better pharmacokinetics while possessing superior thermal stability and improved refolding, qualities that may be of value in cases where maintenance of the cold-chain of transport is difficult.

## Data Availability Statement

The original contributions presented in the study are included in the article/Supplementary Material, further inquiries can be directed to the corresponding author/s.

## Author Contributions

EG, JL, GA, EW, and PG designed the experiments. EG, JL, GA, EW, CB, and CG performed the experiments. EG and GA wrote the manuscript. All authors analyzed data and edited the manuscript.

## Conflict of Interest

JL, GA, and EG are inventors on a patent application: United States Patent Application 16513881 July 17, 2019 Single Domain Antibodies to Chikungunya Virus. The remaining authors declare that the research was conducted in the absence of any commercial or financial relationships that could be construed as a potential conflict of interest.
