# Supplementary material for: Stabilization of a Broadly Neutralizing Anti-Chikungunya Virus Single Domain Antibody
Source: Front Med (Lausanne). 2021 Jan 28;8:626028. doi: 10.3389/fmed.2021.626028 (PMC7876468; doi:10.3389/fmed.2021.626028)
Supplement: Supplementary file 1 [file Data_Sheet_1.pdf]

Supplemental information

Stabilization of a Broadly Neutralizing Anti-Chikungunya Virus Single Domain Antibody

Jinny L. Liu[1#], Emily M. Webb[2#], Dan Zabetakis[1], Crystal W. Burke[3], Christina L. Gardner[3], Pamela J. Glass[3], Patricia M. Legler[1], James Weger-Lucarelli [2], George P. Anderson[1], Ellen R. Goldman[1*]

Contents:

List of sdAb sequences with theoretical pI 2

Supplemental Table S1 4

Supplemental Table S2 5

Supplemental Figure S1 6

Supplemental Figure S2 7

Supplemental Figure S3 8

Supplemental Figure S4 9

Supplemental Figure S5 10

Supplemental Figure S6 11

Expanded methods 12

List of sdAb sequences. Changes from wild type CC3 are highlighted in red; the hop tail is highlighted in green. After each sequence the theoretical pI and MW (in Da) are listed. The theoretical pI and MW were calculated from the web-based ExPASy Compute pI/Mw tool.

CC3-hop

MAEVQLQASGGGSVQAGGSLRLSCVTSQNLFEYYTMGWYRQVPGSQRERVALINNGG
STVAGSVEGRFTISRDHAKNSVYLQMNYLKPEDSAVYYCRAFGPADYWGQGTQVTVS
SAAAGAGGSGGAPASNRCSQGSCWNLEHHHHHH
Theoretical pI/Mw: 7.75 / 15700.25

CC3-m1hop

MAEVQLVESGGGSVQAGDSLRLSCVTSQNLFEYYTMGWYRQVPGSQRELVALINNGG
STVAGSVEGRFTISRDHAKNSVYLQMNYLQPEDSAVYYCRAFGPADYWGQGTQVTVS
SAAAGAGGSGGAPASNRCSQGSCWNLEHHHHHH
Theoretical pI/Mw: 6.12 / 15744.26

CC3-m2hop

MAEVQLVESGGGSVQAGDSLRLSCVTSQNLFEYYTMGWYRQVPGSQRERVALINNGG
STVAGSVEGRFTISRDHAKNSVYLQMNYLQPEDSAVYYCRAFGPADYWGQGTQVTVS
SAAAGAGGSGGAPASNRCSQGSCWNLEHHHHHH
Theoretical pI/Mw: 6.35 / 15787.29

CC3-m3hop

MAEVQLVESGGGSVQAGGSLRLSCVTSQNLFEYYTMGWYRQVPGSQRERVALINNGG
STVAGSVEGRFTISRDHAKNSVYLQMNYLQPEDSAVYYCRAFGPADYWGQGTQVTVS
SAAAGAGGSGGAPASNRCSQGSCWNLEHHHHHH
Theoretical pI/Mw: 6.63 / 15729.25

CC3-m4hop

MAEVQLVESGGGSVQAGGSLRLSCVTSQNLFEYYTMGWYRQVPGSQRERVALINNGG
STVAGSVEGRFTISRDHAKNSVYLQMNYLKPEDSAVYYCRAFGPADYWGQGTQVTVS
SAAAGAGGSGGAPASNRCSQGSCWNLEHHHHHH
Theoretical pI/Mw: 7.04 / 15729.29

CC3-m5hop

MAEVKLQASGGGSVQAGGSLRLSCVTSQNLFEYYTMGWYRQVPGSQRERVALINNGG
STVAGSVEGRFTISRDHAKNSVYLQMNYLKPEDSAVYYCRAFGPADYWGQGTQVTVS
SAAAGAGGSGGAPASNRCSQGSCWNLEHHHHHH

Theoretical pI/Mw: 8.40 / 15700.30

CC3-m6hop

MAQVKLQASGGGSVQAGGSLRLSCVTSQNLFEYYTMGWYRQVPGSQRERVALINNGG
STVAGSVEGRFTISRDHAKNSVYLQMNYLKPEDSAVYYCRAFGPADYWGQGTQVTVS
SAAAGAGGSGGAPASNRCSQGSCWNLEHHHHHH

Theoretical pI/Mw: 8.75 / 15699.31

CC3-m7hop

MAQVKLQASGGGSVQAGGSLRLSCVTSQNLFEYYTMGWYRQVPGSQRERVALINNGG
STVAGSVVGRFTISRDHAKNSVYLQMNYLKPEDSAVYYCRAFGPADYWGQGTQVTVS
SAAAGAGGSGGAPASNRCSQGSCWNLEHHHHHH

Theoretical pI/Mw: 9.00 / 15669.33

CC3-m8hop

MAQVKLQASGGGSVQAGGSLRLSCVTSQNLFEYYTMGWYRQVPGSQRERVALINNGG
STVAGSVKGRFTISRDHAKNSVYLQMNYLKPEDSAVYYCRAFGPADYWGQGTQVTVS
SAAAGAGGSGGAPASNRCSQGSCWNLEHHHHHH

Theoretical pI/Mw: 9.18 / 15698.37

CC3-m9hop

MAQVKLQASGGGSVQAGGSLRLSCVTSQNLFEYYTMGWYRQVPGSQRERVALINNGG
STVAGSVKGRFTISRDHAKNSVYLQMNYLKREDSAVYYCRAFGPADYWGQGTQVTVS
SAAAGAGGSGGAPASNRCSQGSCWNLEHHHHHH

Theoretical pI/Mw: 9.35 / 15757.44

CC3-m10hop

MAQVKLQASGGGSVQAGGSLRLSCVTSQNLFEYYTMGWYRQVPGSQRERVALINNGG
STVAGSVKGRFTISRDHAKNSVYLQMNYLKRADSAVYYCRAFGPADYWGQGTQVTVS
SAAAGAGGSGGAPASNRCSQGSCWNLEHHHHHH

Theoretical pI/Mw: 9.51 / 15699.40

Supplemental Table S1. Neutralization of CHIKV 181/25 by the five originally reported anti-CHIKV sdAb.

CHIKV SdAb	CHIKV $PRNT_{50}$ (µg/mL)	CHIKV $PRNT_{90}$ (µg/mL)
CC3	0.0024	0.0118
CA6	0.3106	1.4500
CH5	0.6300	2.2000
CH6	8.1000	>80
CD11-1	34.9000	>80

Supplemental Table S2. Yields, melting temperature and refolding of variants expressed without the hop tail

Clone	Theoretical pI	Yield mg/L	Tm (CD)	% refold
CC3	7.05	10	60	62
CC3-m1	5.91	15	75	95
CC3-m2	6.12	7.5	67	95
CC3-m3	6.35	9	71	91
CC3-m4	6.64	12.9	70	83
CC3-m5	7.86	15	59	62
CC3-m6	8.62	7.5	57	46
CC3-m7	9.00	7.2	56	45
CC3-m8	9.23	15.3	58	31
Cc3-m9	9.43	2.2	53	9
Cc3-m10	9.59	1.7	43	7

Supplemental Figure S1

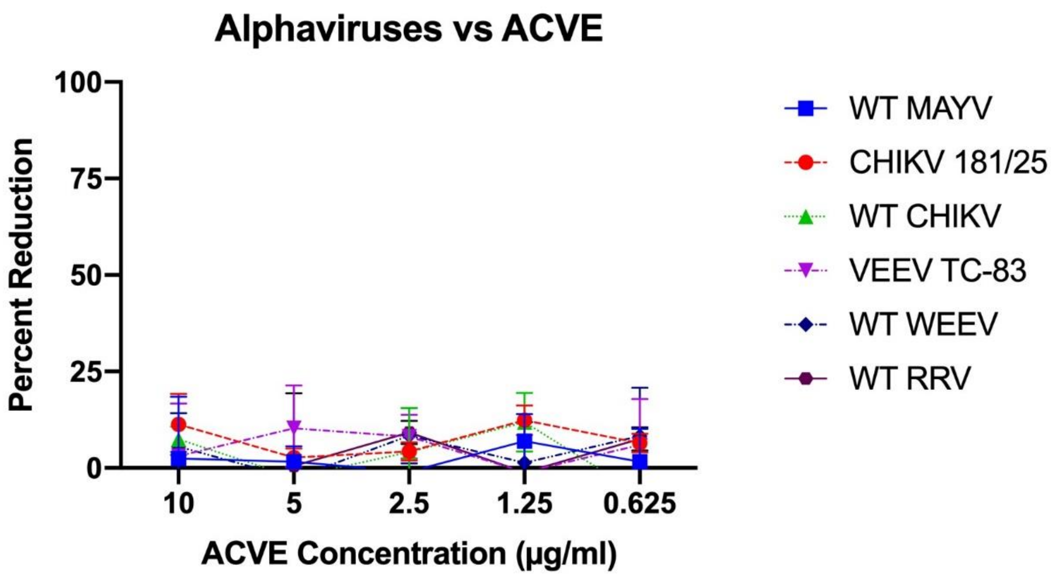

Isotype control. Ability of toxin binding sdAb ACVE to neutralize the indicated alphaviruses. No neutralization was observed.

Supplemental Figure S2

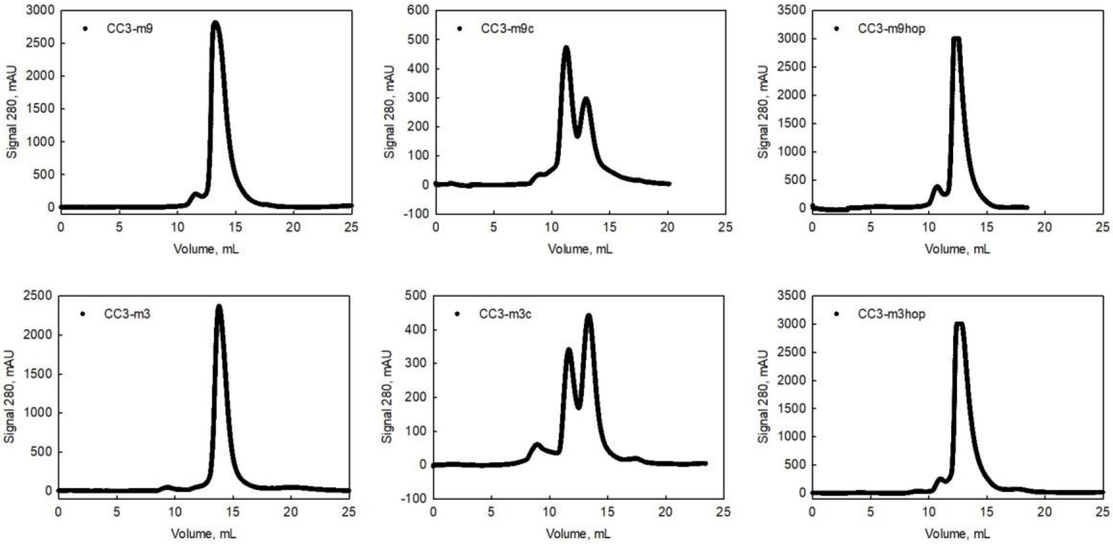

Profiles off the size exclusion column that was run after the immobilized metal affinity chromatography step of the protein purification. Two of the variants (CC3-m9 and CC3-m3) are shown; all of the variants showed essentially the same pattern. The left panels are constructs with only a 6-histidine tail, the middle panels show the constructs with the C-terminal cysteine, and the right panels show constructs with the hop tail. The monomeric peak was collected and used in the characterization of the variants. Because of the low yield of the variants with the C-terminal cysteine, only four these preparations were run on the gel filtration column, and none were characterized further.

Supplemental Figure S3

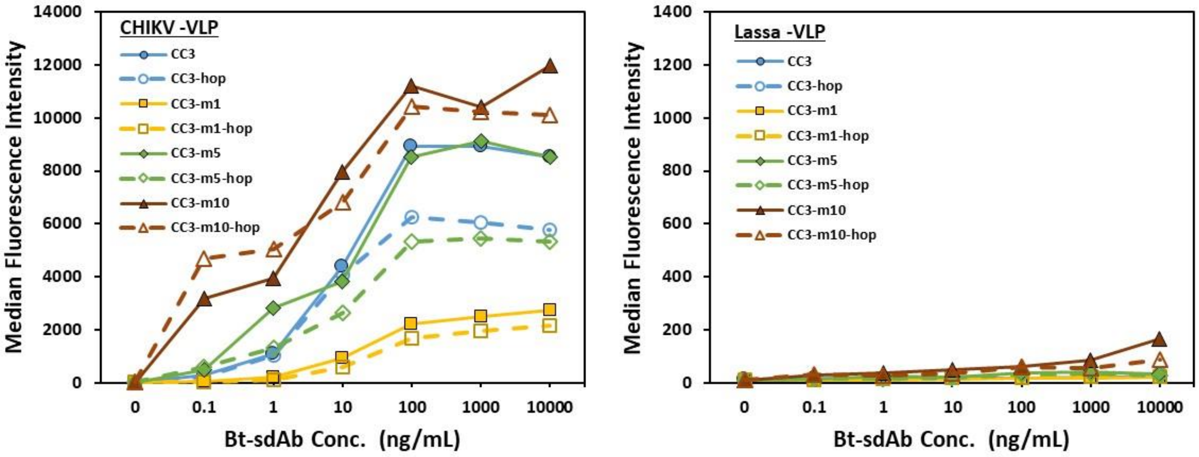

MagPlex direct binding data. Left panel compares the ability of CC3 variants with and without the hop tail to bind CHIKV VLPs immobilized on magnetic microspheres. For these experiments, the sdAb were all biotinylated through primary amines, and not through their cysteine-containing hop tail. Differences in signal magnitude at the higher concentrations in the case of CC3-m3 and CC3-m5 may be attributed to the extent of biotinylation in the preparations with and without the hop tail. These experiments verified that addition of the hop tail did not obviate binding ability. Right panel shows the ability of the CC3-hop variants to bind an irrelevant viral target, Lassa VLPs immobilized on magnetic microspheres. Minimal non-specific signal was observed for CC3-m10, the most positively charged mutant and for CC3-m10hop. Please note the scale of the Y axis in the right panel is an order of magnitude lower than in the left panel.

Supplemental Figure S4

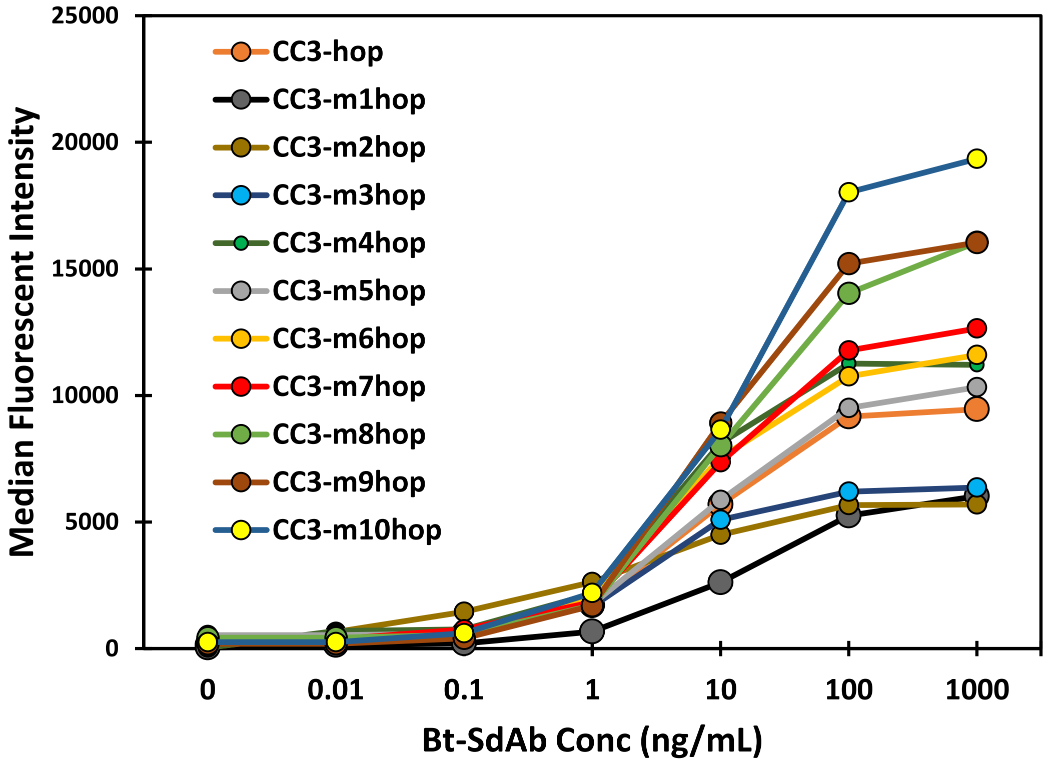

MagPlex direct binding data showing the ability of the CC3-hop variants to bind CHIKV VLPs immobilized on magnetic microspheres. For these experiments, the sdAb were all biotinylated through primary amines, and not through their cysteine-containing hop tail.

Supplemental figure S5

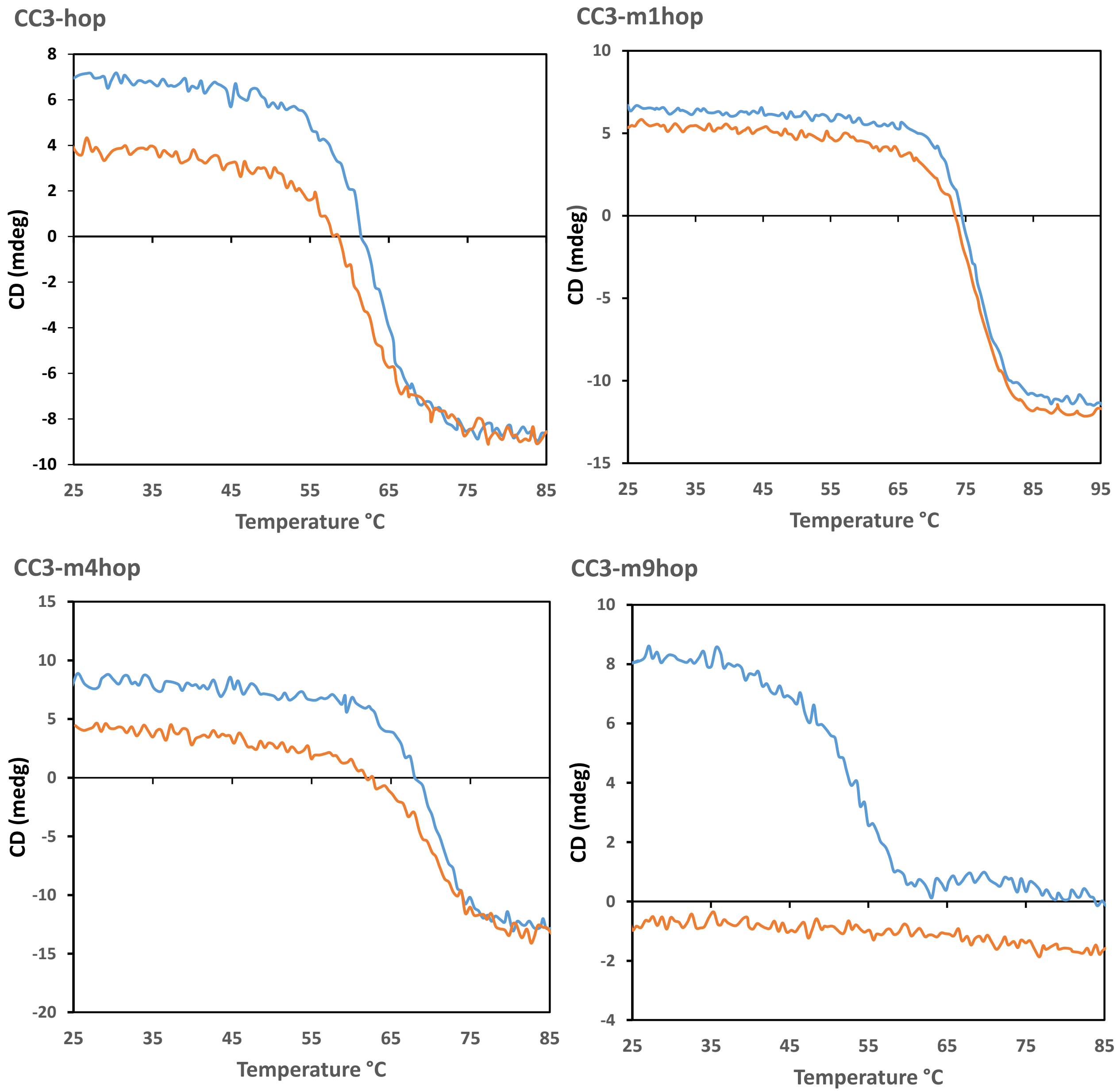

Circular Dichroism data showing melting (blue) and refolding (orange) curves for the CC3-hop (top left) and the indicated CC3-hop variants.

Supplemental figure S6

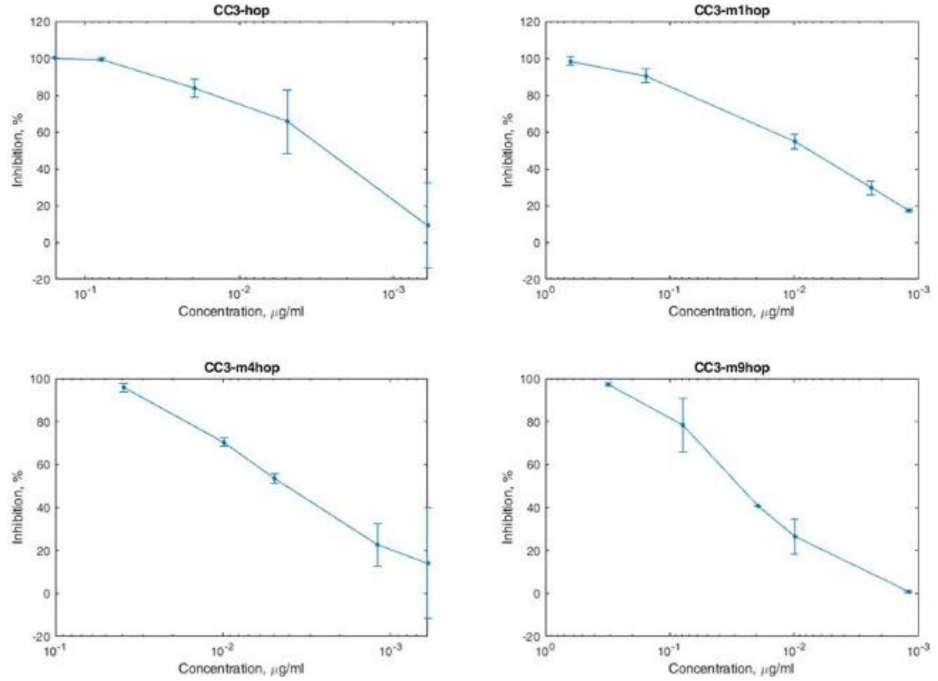

Neutralization curves for several representative CC3 derivatives were plotted by selecting data points within the range of neutralizing concentrations. All the data points were obtained from duplicate measurements and error bars represent standard deviation.

Expanded methods

Construction of sdAb variants

Genes were synthesized by Eurofins Genomics (Louisville, KY). The sdAb variants were synthesized with flanking NcoI and NotI sites, and the hop tail was synthesized with flanking NotI and XhoI sites. Genes were digested with the appropriate restriction enzymes and purified by gel electrophoresis and the qiagen QIAquick gel extraction kit. First the hop tail was ligated into pET22b vector digested with NotI and XhoI to create pET22b-hop. Each of the CC3 variants was ligated into both pET22b-hop and pET22b that had been digested with NcoI and NotI.

Choice of mutations

Mutations were chosen by looking at the sequences of published CHIKV binding sdAb that we had isolated (CA6 in particular) as well as the sequences of published and well-producing toxin binding sdAb. The purpose was to find framework positions which included the addition or deletion of charged amino acids from CC3 to create a series of mutants that spanned the range of isoelectric points. In the case of m10, we also looked at the sequence of V3G9, a well expressed (~16 mg/L) unpublished sdAb that binds the Venezuelan Equine Encephalitis virus whose sequence is as follows: EVQLQASGGGLVQAGGSLKV SCAASGRTFNNLAMAWFREAPENEREFVAAIMWTGDRTHYADFVKGRYTISRDNALNTVSLQMNNLKPAD TAVYYCAGAFSFPSQFARDYTYWGQGTQVSVSS. Our rational in choosing the specific mutations was to find framework substitutions that were present in at least one well producing sdAb or that were present in at least one CHIKV neutralizing sdAb. The Q5V and A6E mutations were the only ones which we had previously correlated with an increase in melting temperature.

Production and purification of sdAb constructs

To produce the sdAb, expression plasmids were transformed into the E. coli strain Tuner (DE3). Freshly transformed colonies were used to start cultures (50 mL in terrific broth [TB] with 100 µg/mL ampicillin) which were grown in shake-flasks overnight at 25 °C. The next day, each culture was added to 450 mL of TB with ampicillin and incubated with shaking for 2 hours at 25 °C. Cultures were then induced using isopropyl-D-1 thiogalactoside (IPTG, 0.5 mM) and incubated with shaking for another 2 hours.

Purification of sdAb expressed from pET22b, the periplasmic expression vector, were carried out through an osmotic shock protocol. Cells from each 500 mL shake flask culture were pelleted and resuspended in 14mL of 100mM Tris, 0.75M sucrose pH 7.5. Next 1 mL of lysozyme (1 mg/mL) was added followed by 28mL of 1mM EDTA added drop-wise to the solution while the centrifuge tubes held in crushed ice were gently shaking on a rotating platform. After addition of the EDTA, 0.25 mL of 5% deoxycholate was added and the cells were gently swirled for another half hour. Lastly 1mL of 0.5M $MgCl_2$ was added and incubated for a further 15 minutes and then the spheroplasts were pelleted. The supernatant was poured into a 50 ml conical tube containing ~ 0.5 ml of immobilized metal affinity chromatography (IMAC) resin (Ni Sepharose High Performance, GE Healthcare, Marlborough, MA, USA) and 5 mL 10x IMAC buffer (0.2 M Na_2HPO_4, 4 M NaCl, 0.25 M imidazole pH 7.5 plus 0.02% sodium azide). The mixture tumbled for two hours at 4°C and then was washed twice in batch with 1x IMAC (0.02 M Na_2HPO_4, 0.4 M NaCl, 0.025 M imidazole pH 7.5 plus 0.002% sodium azide). The next day the sdAb was eluted from the resin with 1x IMAC containing 250 mM imidazole and further purified by fast protein

liquid chromatography (FPLC) using a Bio-Rad Duo-flow System with a Enrich SEC 70 10x300 mm column (Bio-Rad, Hercules, CA, USA) equilibrated with phosphate buffered saline (PBS) with 0.02% sodium azide. Concentration and yields were determined from the absorbance at 280 nM. Samples were stored either at 4°C for immediate use or at -80 °C for long term storage.

Circular Dichroism

Circular dichroism (CD) was performed using a Jasco J-815 Spectropolarimeter. The sdAb samples were diluted to 22 μg/mL in deionized water and placed in a quartz cuvette with 1 cm path length and CD was measured at an ultraviolet wavelength between 200 and 210 nm. The sdAb samples were heated from 25°C to 85 or 95°C at a rate of 2.5°C/min and then cooled back to 25°C at the same rate.

MagPlex Direct Binding Assays

Specificity and an indication of affinity was appraised via the direct binding of the sdAb to CHIKV VLPs immobilized on MagPlex magnetic microspheres (Luminex, Austin, TX, USA). The CHIKV VLPs along with unrelated proteins were immobilized to unique sets of MagPlex microspheres (30 µL each) using a standard EDC (1-ethyl-3-(3-dimethylaminopropyl)carbodiimide hydrochloride) and sNHS (N-hydroxysulfosuccinimide) immobilization protocol, provided by the manufacturer, where after the microspheres are washed three times using 0.1 M Sodium Phosphate pH 6.0 (wash buffer) and resuspended in 100 µL of that buffer and then activated using 100 µL a mixture of 1:1 EDC 0.4 M and sNHS 0.1 M. After 20 minutes the microspheres are washed with the wash buffer 2 times and once with PBS before addition of the 5 to 40 μg of the protein to be immobilized.

To prepare the biotinylated sdAb a 10-fold excess of EZ-Link NHS-LC-LC-Biotin (Thermo Fisher Scientific) was used, each of the sdAb was biotinylated for 30 minutes; excess biotin was removed using Zeba spin columns (Thermo Fisher Scientific). The absorbane at 280 nM was used to calculate the concentration of biotinylated sdAb (Bt-sdAb). Dilutions of each Bt-sdAb in PBSTB (phosphate buffered saline [PBS] + 0.05% Tween+ 0.1% bovine serum albumin [BSA]) were prepared in round bottom polypropylene microtiter plates (VWR). The mixture of antigen-coated MagPlex microspheres was added to the wells and gently shaken in the dark for 30 minutes to allow the binding to occur. Afterwards the plate was washed with PBST and then incubated with 5 μg/mL streptavidin-conjugated phycoerythrin (SA-PE) for 30 min, washed, and binding evaluated on the MAGPIX instrument (Luminex).

PRNT

Virginia Tech's PRNT protocol:

Two-fold serial dilutions were prepared with 75μl of CC3 in sdAb diluent (e.g. RPMI-1640 media containing 25 mM HEPES, 1% BSA, 50 μg/mL Gentamicin, and 2.5 μg/mL Amphotericin B) starting at a concentration of 10μg/ml. Each dilution was mixed with 75μl of 1200 PFU/mL alphavirus stock, in triplicate, and incubated at 37°C for one hour. The sdAb/alphavirus mixtures were then added to a monolayer of Vero 76 cells seeded in 24-well plates. Culture plates were incubated for one hour at 37°C, while being rocked every ten minutes, prior to addition of a 1.5% methylcellulose overlay. Plates were incubated at 37°C in a humidified 5% CO_2 atmosphere for 2-4 days (dependent on alphavirus/virus strain). On the last day of incubation, cells were fixed with 10% formalin prior to being stained with crystal violet.

NRL PRNT protocol:

Twelve 2-fold serial dilutions of each sdAb were prepared. The initial starting concentration of sdAb was set at 20 μg/mL. Each dilution was incubated with ~300 plaque forming units (PFU) of virus at 4° C overnight. Each sdAb-virus mix was then split and added to Vero cells seeded in 6-well culture plates for 1 1/2 hour incubation, followed by adding 0.6% agarose overlay in 1XBME (Thermofisher) to each well and incubating for 24 hours at 37 °C in a humidified 5% CO2 atmosphere before the addition of the second 0.6 % agarose overlay containing neutral red in 1xBME. After 24 hours of neutral red staining, the transparent plaques were counted. 50% and 90% plaque reduction neutralization titer (PRNT50 and PRNT90) of each sdAb was calculated using XLfit dose response model. If needed the starting concentration was adjusted to include the dilutions ranging between PRNT50 and PRNT90.
